# Research Progress and Development Trends of Acoustic Metamaterials

**DOI:** 10.3390/molecules26134018

**Published:** 2021-06-30

**Authors:** Hao Song, Xiaodong Ding, Zixian Cui, Haohao Hu

**Affiliations:** 1 Systems Engineering Research Institute, Beijing 100036, China; dingxiaodong@126.com (X.D.); cuizixian@126.com (Z.C.); 2 School of Navel Architecture and Ocean Engineering, Jiangsu University of Science and Technology, Zhenjiang 212000, China; 18751589046@163.com

**Keywords:** functional materials, acoustic metamaterials, acoustic metasurface, meta-atom, metamolecule

## Abstract

Acoustic metamaterials are materials with artificially designed structures, which have characteristics that surpass the behavior of natural materials, such as negative refraction, anomalous Doppler effect, plane focusing, etc. This article mainly introduces and summarizes the related research progress of acoustic metamaterials in the past two decades, focusing on meta-atomic acoustic metamaterials, metamolecular acoustic metamaterials, meta-atomic clusters and metamolecule cluster acoustic metamaterials. Finally, the research overview and development trend of acoustic metasurfaces are briefly introduced.

## 1. Introduction

Waves are a ubiquitous form of motion in nature, and the research on wave regulation (including wave propagation direction and physical properties, etc.) not only has a wide range of applications, but also greatly promotes the development of science and technology. There are many materials in nature that can control waves (acoustic waves, electromagnetic waves, etc.), and the response parameters of materials are all positive values. To break through the regulation of waves by conventional materials in nature, functional materials have been introduced with properties that can be significantly changed in a controlled fashion by external stimuli. Acoustic metamaterials (AM) and phononic crystals have attracted the attention of researchers, and great progress has been made in engineering applications.

In 1968, the Soviet physicist Veselago [1] proposed the concept of left-handed materials, and it was later verified theoretically and experimentally by Pendry et al. [2,3] and Smith et al. [4]. These types of materials opened up a new way to control electromagnetic waves and other forms of waves. The electromagnetic metamaterial developed from the left-handed material is an artificially designed material that has negative refraction, anomalous Doppler, anomalous Cherenkov radiation, perfect lens, invisibility and other abnormal control effects on electromagnetic waves [5,6,7,8,9].

Since both electromagnetic waves and acoustic waves meet the relevant properties of fluctuations, have common wave parameters, (such as wave vector, wave impedance, and energy flow), and both satisfy the wave equation; researchers have extended the design ideas of electromagnetic metamaterials to the field of acoustics. In this paper, we first review the research advances in AM in recent 20 years and then mainly discuss the properties of the meta-atom AM (MAAM), metamolecule AM (MMAM), meta-atom cluster AM, and metamolecule cluster AM.

The MAAM consists of local resonant meta-atoms, which resonant frequency is related to the size of the structure. The MAAM presents the transmission dip and inversed phase near the resonant frequency. The meta-atoms discussed in this paper contain the split hollow sphere (SHS) and hollow tube (HT), which can be used to realize the AM with single-negative modulus and AM with single-negative mass density near the frequency, respectively. Furthermore, by coupling the two kinds of meta-atoms in a structure, a “flute-like” metamolecule structure of perforated hollow tube can be realized. This can be used to fabricate double-negative AM in high or low frequency band.

The meta-atom cluster AM can be fabricated by arraying different sized meta-atoms. The meta-atom cluster AM composed of different sized meta-atoms of SHSs can realize multi-band or broadband negative modulus, and the different sized meta-atoms of HTs can realize broadband negative mass density. Similarly, the metamolecule cluster AMs are constructed with seven kinds of “flute-like” perforated hollow tubes, which can overcome the limitations of arbitrary broadband negative bulk modulus and mass density to provide a region of inverse Doppler effects.

As the resonant unit can realize the effect of discontinuous phase, it can be used to design acoustic metasurface (AMS) to control the acoustic wavefronts at will and to realize the anomalous manipulation of acoustic waves. Finally, the research status and tendency of AMS in coming years are introduced.

## 2. Research Progress of Acoustic Metamaterials

### 2.1. Overview of the Development of Acoustic Metamaterials

In 2000, Liu et al. [10] first proposed the use of local resonance type structural units to construct AM. This idea opened up a new pathway for the acoustic research. Similar to the research method of electromagnetic metamaterials [2,3,4,11,12], The focus was on how to achieve negative mass density elastic modulus, double-negative AM, and many theoretically models were proposed [13,14,15].

In 2008, Yang et al. [16] experimentally proposed and prepared a two-dimensional film-mass structure of negative mass density AM, and systematically studied the singular properties of AM based on this structure [17,18]. At the same time, researchers have implemented negative mass density AM through many methods [18,19,20,21,22,23,24,25,26,27], and broadened the range of acoustic materials by studying anisotropic mass density materials [28,29,30,31,32]. In 2006, Fang et al. [33] proposed an ultrasonic metamaterial composed of a sub-wavelength scale one-dimensional Helmholtz resonant cavity array and a propagation channel. This material has a negative elastic modulus near the resonance frequency. Inspired by Fang et al., researchers have proposed a variety of negative equivalent elastic modulus models. The Helmholtz resonator model can be extended to two-dimensional and three-dimensional situations, and negative elastic modulus AM can also be obtained [34,35,36,37]. By opening holes in the side wall of the hollow tube (HT) [38], an AM with a negative equivalent elastic modulus of propagation cut-off frequency can be realized. Ding et al. [39,40,41,42,43,44] proposed an open hollow sphere, where the structure realizes the negative equivalent elastic modulus in the air medium. Leroy et al. [45] realized the negative elastic modulus through the bubble array. Compared with the single-negative AM, the elastic modulus and mass density are both negative at the same time. Negative AM have more exotic properties [46]. Double-negative metamaterials are mainly realized by combining two single-negative MA structures. Ding et al. [47] proposed a zinc-blende structure, consisting of a water ball-coated bubble structure and a rubber-coated gold ball in an epoxy resin. Theoretically, it can simultaneously achieve a negative equivalent mass density and a negative equivalent elastic modulus. Lee et al. [48] combined a HT structure with periodically arranged films and a tubular Helmholtz resonator structure with periodic holes on the sidewall to realize a double-negative AM, and tested its anomalous Doppler Effect [49]. Chen et al. [50,51,52,53] presented the combination of a HT structure with negative mass density and an open hollow sphere structure with negative elastic modulus which can realize double-negative acoustic metamaterials, and at the same time, the two structures can be coupled into a metamolecule to achieve double-negative AM. Fok and Zhang [54] proposed to couple a Helmholtz resonator and a plexiglass-clad aluminum column in the same aluminum body cavity to prepare double-negative and negative refractive index acoustic metamaterials. However, if there are two resonance modes in a single structural unit, double-negative metamaterials can also be realized. Yang et al. [55] designed a double-film system, which achieved double-negative acoustic parameters in the range of 520–830 Hz by adjusting the resonant frequencies of monopole resonance and dipole resonance. Lai et al. [56] designed an elastic AM based on a solid substrate, which can realize two double-negative dispersion bands. Pope and Daley [57] proposed a viscoelastic double-negative AM theoretical model, whose negative dynamic mass density and elastic modulus can be tuned.

AM designed by MA and metamolecules (MM) have many unique properties [58,59,60], including flat panel focusing, negative refraction, subwavelength imaging, stealth, anomalous Doppler effect, abnormal sound transmission, etc. Unlike phononic crystals [61,62], AM are based on the principle of resonance to achieve negative refraction focusing. Based on the acoustic transmission line model [63,64], the combination of two Helmholtz resonators can achieve ultrasonic focusing in water. The Helmholtz resonator or labyrinth-like structure is designed as a two-dimensional AM [65,66], and the negative refraction effect has been realized experimentally. García-Chocano et al. [67] used a hyperbolic metamaterial to achieve the negative refraction effect. Xia and Sun [68] designed a non-resonant ring structure, through its natural mode at a specific eigenfrequency, to achieve the focus of the sound wave at the center of the ring structure. Zhai et al. [52] realized the negative refraction of the audible sound frequency band in air medium through a wedge-shaped sample composed of a drilled HT.

Similar to the principle of surface plasmon amplifying evanescent wave in the electromagnetic field [69,70,71], in the field of acoustics, the use of negative equivalent mass density AM can amplify evanescent waves [72] and acoustic near-field super-lens for super-resolution imaging [73]. There are other ways to implement acoustic super-lens; Zhu et al. [74] used the Fabry-Perot resonance coupling generated by the periodically arranged hole structure to amplify the evanescent wave and achieved near-field super-resolution imaging (λ/50). Kaina et al. [75] used a single-negative metamaterial prepared by a single resonator to achieve negative refractive index acoustic superlens. Similar to the design method of the electromagnetic far-field lens (hyperlens) [76,77,78], the use of AM can also realize the far-field amplification of evanescent waves [79,80,81,82], and realize the acoustic far-field super-resolution lens. Li et al. [83] proposed a two-dimensional acoustic far-field hyperlens based on a fan-shaped structure, which can achieve sub-wavelength far-field super-resolution imaging of broadband sound waves with a resolution of λ/6.8—λ/4.1.

AM can also be used to design perfect acoustic absorbers [84]. In 2010, Pai [85] theoretically proposed a broadband elastic wave absorber, which made it possible to completely absorb sound waves. Mei et al. [86] used the resonance of a thin film based on an additional metal sheet to achieve broadband sound absorption in low frequency domain of 100–1000 Hz, and the sound absorption efficiency reached 86% at 172 Hz. After the film is made into a double layer, the sound absorption rate reached 99% at certain frequencies. Ma et al. [87] designed a narrow-frequency selective filter with a combination of a thin film structure and an air channel. In recent years, researchers have used many methods to design AM to achieve high-efficiency absorption of sound waves [88,89,90,91].

Based on the electromagnetic cloak design method proposed by Pendry et al. [92,93], Chen and Chan [94] proposed a spherical Bessel function system expansion method to solve the sound scattering problem, and designed a three-dimensional acoustic cloak [95,96]. The transformation acoustic formula is improved, and in theory, the multi-layer concentric column structure can be used to achieve acoustic stealth [97,98,99]. Since stealth materials require very high material parameters and are difficult to prepare for experiments, Zhang et al. [100] overcame the above problems by introducing acoustic transmission line theory. A two-dimensional cylindrical cloak was designed to achieve 52–64 kHz wideband ultrasonic stealth. Zhu et al. proposed that acoustic stealth can also be achieved through single-negative metamaterials [101]. In order to avoid complicated parameter design, the cloak is designed into a rhombus structure, and only a uniform medium can be used to achieve acoustic stealth [102,103]. On this basis, Zigoneanu et al. [104] realized a nearly perfect three-dimensional, broadband, and all-round three-dimensional carpet invisibility cloak through theoretical design and experiments. In 2006, Hu et al. [105] experimentally realized the anomalous Doppler effect in the band gap of a phononic crystal. Lee et al. [49] used the designed double-negative acoustic metamaterial to achieve this anomalous Doppler effect in the same experiment. Zhai et al. [106] used AM prepared by MM clusters to achieve a broadband anomalous Doppler effect.

AM can also be used to achieve anomalous acoustic transmission effects [107,108,109], long-distance acoustic collimation of long-wavelength sound waves [110], and design of acoustic diodes, to realize the non-conducting and easy propagation of sound wave energy [111,112,113,114,115,116]. In just a dozen years, AM have been developed rapidly, producing many new unique properties, and have been applied to many fields, such as ultrasonic imaging, underwater acoustics and sonar, architectural acoustics and sound-absorbing materials, etc. [117,118,119].

### 2.2. Meta-Atomic Acoustic Metamaterials (MAAM)

#### 2.2.1. Negative Elastic Modulus MA

**Figure 1:**
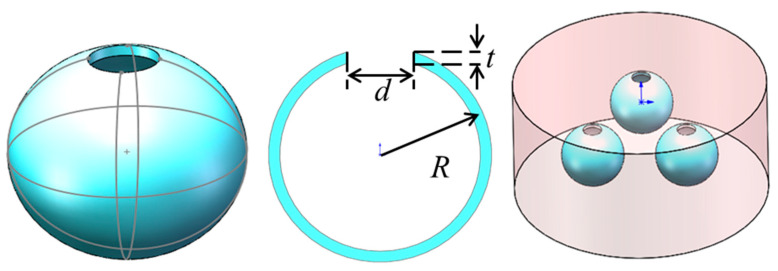
The schematic diagram of SHS unit cell.

The singular properties of AM are mainly realized by designing suitable artificial acoustic MA. In this field, split-ring resonators (SRRs) have local resonance properties, and can be used to prepare negative permeability materials [3]. In addition, a locally resonant MA split hollow sphere (SHS) structural unit could also be realized [39,40,41,42,43,44]. The SHS shown in Figure 1 is a hollow sphere with a hole of a certain diameter. The body cavity of the SHS can store sound energy. The opening will cause the acoustic medium to vibrate in and out. When the resonance frequency is reached, the energy accumulated in the body cavity causes the acoustic medium to vibrate strongly at the opening to achieve resonance, and the resonance unit is the basic meta-atom for the preparation of AM. Its resonance frequency could be expressed as:

(1) $$f_{0}=\frac{1}{2\pi \sqrt{L_{0}C_{0}}}=\frac{C_{0}}{2\pi \sqrt{\frac{Vd_{eff}}{S_{1}}}}$$

where $S_{1}=\pi {\left(\frac{d}{2}\right)}^{2}$ is the cross-sectional area of the opening; *V* is the volume of the hollow sphere of SHS; and $\rho_{0},C_{0}$ are the density and sound velocity of air. At resonance, the sound radiation at the opening will generate radiation impedance, the equivalent length of the open tube is increased, and after correction $d_{eff}=t+1.8\sqrt{d}$.

#### 2.2.2. Negative Mass Density MA

**Figure 2:**
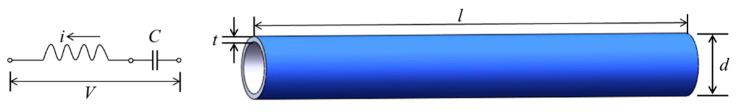
The unit cell of hollow steel tube (HT) structure.

In analogy electromagnetics, the metal rod array [2] realizes the equivalent dielectric constant ε<0. In the field of acoustics, a HT artificial MA resonance model that can achieve negative mass density is proposed [26,50], as shown in Figure 2. HT is a hollow steel tube structure with openings at both ends. Cylindrical HT with openings at both ends have a guiding effect on sound waves. This structure can be equivalent to the inductance of the acoustic circuit $L=\rho_{0}l/S$, where *S* is the cross-sectional area of the port, *l* is the aperture length.

The inside of the HT can be regarded as a kind of body cavity, which has the function of storing sound wave energy, which is equivalent to the function of sound volume, so the equivalent sound volume, $C=V/\rho_{0}C_{0}^{2}$ where V is the volume of the cavity, and $C_{0}$ is the speed of sound in the fluid, $\rho_{0}$ is the density of the background fluid. The resonant frequency calculated based on the L-C resonance model is:

(2) $$f_{0}=\frac{1}{2\pi \sqrt{LC}}$$

#### 2.2.3. Double-Negative MA

**Figure 3:**
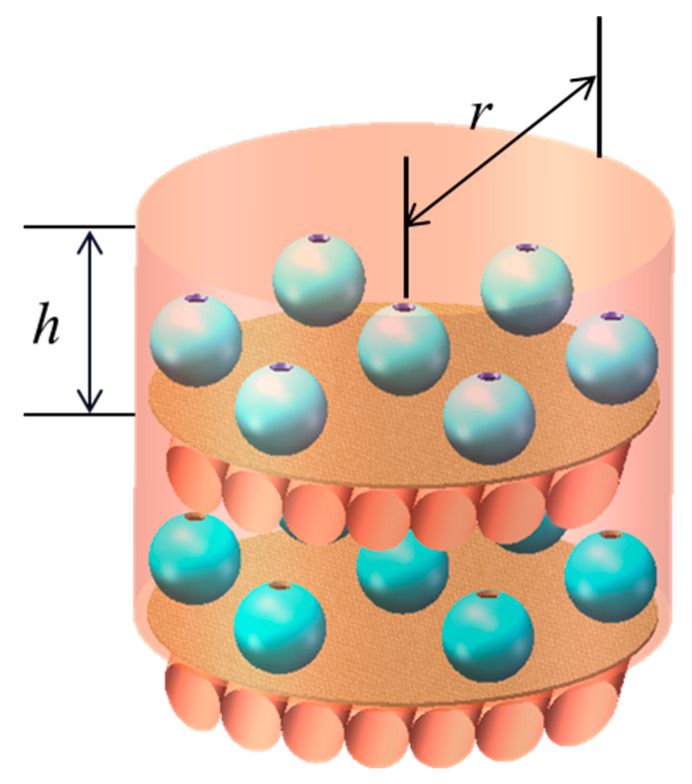
The double-negative acoustic metamaterial: The schematic diagram of the sample.

Similar to the combination of the open metal ring and the metal rod structure to prepare the electromagnetic left-handed material, the HT structure and the SHS structure are superimposed to form a double-layer SHS and a double-layer HT model to make a double-negative AM [50], such as shown in Figure 3.

### 2.3. Metamolecules Acoustic Metamaterials (MMAM)

MM can be formed by the integration of two MA, and a double-negative AM can also be realized through an MM structure [118,119]. The HT-MA with negative mass density and the open hollow sphere MA with negative elastic modulus are fused together, and a HT structural unit with side holes can be designed. This is also known as a “flute-like” acoustic MM structure. Using this structural unit, a double-negative AM was realized at low and high frequency respectively [51,52], and its acoustic singular properties were studied.

**Figure 4:**
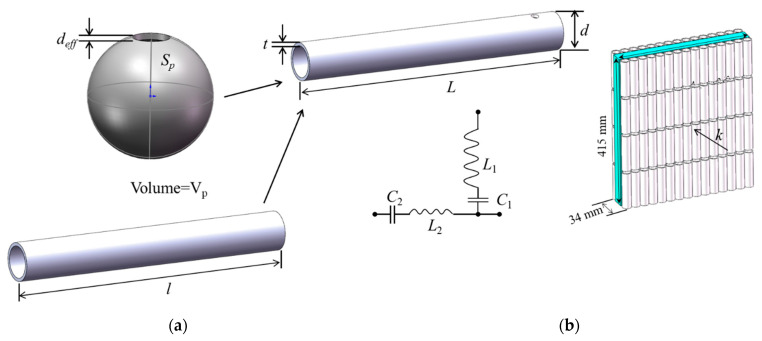
Flute-like metamolecule acoustic metamaterial: (**a**) The structure of metamolecule; (**b**) the schematic diagram of the sample.

As shown in Figure 4, both the open hollow sphere and HT-MA are sub-wavelength local resonant structural units, which can be equivalent to L-C oscillator circuits. The air flow inside the structural unit can be regarded as the charge flow in the oscillating circuit. For HTs, air enters and exits through the two ports of the HT, which compresses and expands the fluid enclosed in the cavity. Therefore, the port of the tube can be regarded as the sound perception *Lt* in the acoustic circuit, and the cavity of the tube can be regarded as the sound capacity $C_{t},L_{t1}=\rho_{0}L_{t1}/S_{t1},L_{t2}=\rho_{0}L_{t2}/S_{t2}$, $C_{t}=V_{t}/\left(\rho_{0C_{0}^{2}}\right)$, where $S_{t1}=S_{t2}$, is the cross-sectional area of the cavity at the end of the plastic tube; $L_{t1}$ and $L_{t2}$ are the equivalent lengths of the two ends, respectively; *V_t_* is the volume of the tube cavity; $\rho_{0}$ is the density of the fluid; $C_{0}$ Is the speed of sound in the fluid. SHS is equivalent to a Helmholtz resonator. The opening and internal cavity of SHS are equivalent to inductance *L_p_* and capacitance *C_p_*, respectively. MM model can be seen as an SHS embedded in a HT. According to the L-C oscillator circuit described in the figure, integrating L and C together, the resonance frequency can be written as:

(3) $$f=\frac{1}{2\pi \sqrt{L_{eff}C_{eff}}}$$

where $L_{eff}=L_{t1}+L_{t2}+L_{p}$, $C_{eff}=\frac{C_{t}C_{p}}{C_{t}+C_{p}}$.

MM unit prepared in the experiment is a hollow plastic tube with side holes. The structural units are arranged periodically in a “Z” shape according to the opening positions, and fixed on the front and back sides of the sponge substrate with adhesive to prepare a double-layer metamaterial sample, as shown in Figure 4b.

### 2.4. Meta-Atomic Clusters and Metamolecular Cluster Acoustic Metamaterials

**Figure 5:**
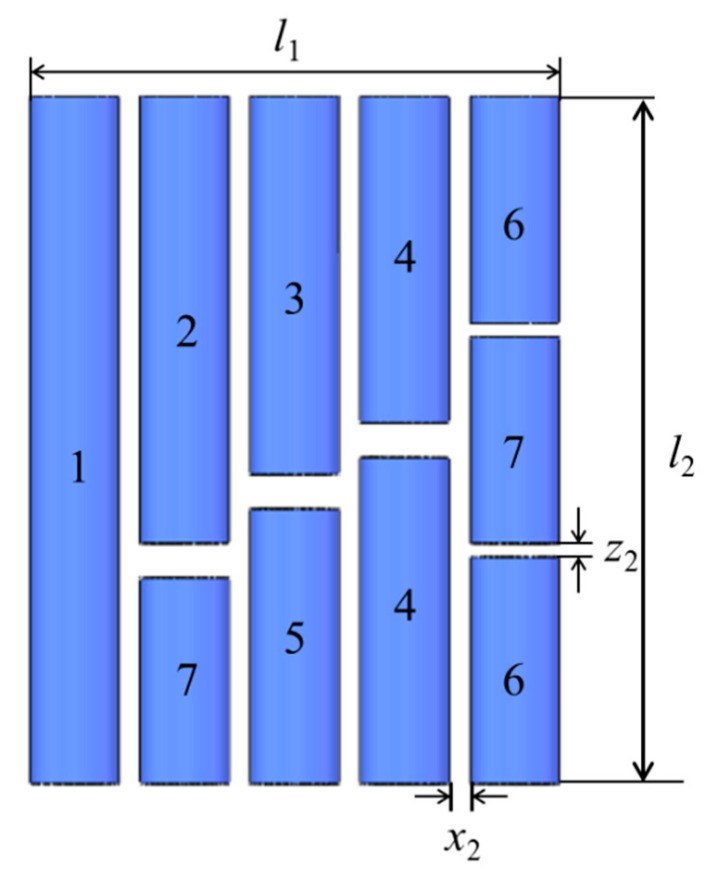
The scheme of the MA cluster acoustic metamaterial structure.

Previous studies have shown that the acoustic behavior of the periodically arranged single HT-MA is basically not affected by the surrounding MA, and there is also a weak interaction between them. Using the behavior of the HT-MA, HT-MM clusters of different lengths could be developed. AM are prepared by periodically arranging metamolecular clusters in a sponge substrate [26], as shown in Figure 5. Based on the weak interaction properties, SHS structures with close apertures are combined into MA clusters, and it is possible to design a broadband 900–1500 Hz negative elastic modulus acoustic metamaterial [40].

The “flute-like” acoustic metamolecules also have weak interactions. Seven types of metamolecular clusters are combined into AM [105]. Simulation calculations and experiments have confirmed that this local resonance elastic modulus and mass density are double-negative AM, and can achieve a negative refractive index of the material at a wide frequency. Tests have also shown that this metamaterial exhibits a broadband anomalous Doppler effect, and as the frequency increases, the frequency shift value continuously increases. In theory, MM clusters can be used to assemble any broadband double-negative acoustic metamaterials, which also opens up new ways for the design and various applications of acoustic metamaterials.

## 3. Progress in Acoustic Metasurface Research

At the end of 2011, the interfacial phase discontinuity theory was proposed [120], and metasurface materials were introduced [121]. This material interface phase is discontinuous, which can arbitrarily adjust the phase distribution according to the geometric size of the structure, thereby adjusting the electromagnetic wave propagation [122,123,124,125,126,127]. The design concept was quickly introduced into the field of acoustics, using acoustic metasurfaces (AMS) to achieve subjective control of the sound wave propagation path [58].

In 2013, Li et al. [128,129] designed a two-dimensional ultra-thin AMS using a curly space structure, which achieved arbitrary control of reflected sound waves theoretically and experimentally. The structural unit is along the propagation direction of the sound wave. The overall thickness was only 1 cm, which is much smaller than its working wavelength (19.0 cm). Zhu et al. [130] proposed a dispersion-free wavefront modulation method, and designed a sub-wavelength fold-shaped surface composed of 18 grooves with different depths, which can achieve adjustment of reflected sound waves in a wide frequency range. Ding et al. [131,132,133] designed an AMS using an open hollow sphere structure with a negative equivalent elastic modulus. This basic structural unit has good coupling and tuning. The resonance frequency can be adjusted only by adjusting the opening diameter of the open hollow ball. The resonance frequency ranges between 0–2 π. Simulations and experiments have confirmed that this structure can be used to control the propagation phase of sound waves, and can realize the abnormal reflection of sound waves. Zhao et al. [134,135] concluded that it can also control the propagation phase of sound waves by changing the impedance at the interface, so as to achieve abnormal reflection of sound waves.

In addition to abnormal reflections, AMS can also achieve abnormal refraction of transmitted waves. The method of using MS to control transmitted waves is similar to that of reflected waves. By adjusting the propagation phase of the transmitted waves, arbitrary control of the propagation direction of the transmitted waves can be achieved, and basic requirements are required. The transmission efficiency of the unit should be as large as possible, so that the acoustic metasurface designed by the basic unit can ensure the high-efficiency abnormal regulation of the transmitted wave; in recent years, many researchers have begun to try to use the acoustic metasurface to achieve abnormal transmission. Xie et al. [136] designed an acoustic metasurface through a spiral labyrinth structure, the overall thickness of which is about half of the working wavelength, which can achieve obvious abnormal refraction. Tang et al. [137] used the optimized labyrinth structure to design and prepare an acoustic metasurface with a thickness of only 1/6.67 of the working wavelength, and realized the abnormal regulation of the 2.25 kHz transmitted sound wave with high efficiency. Mei and Wu [138] adjusted the phase of the structural unit by changing the refractive index, and also achieved arbitrary control of the transmitted sound wave. Zhu and Semperlotti [139] designed a basic unit that can accurately control the phase of incident sound waves by using a local resonance ring cone. Using basic units to construct phase-discontinuous AMS, it can control the abnormal refraction of the elastic guided wave mode in the thin-walled structure. The drum-like structure designed by Zhai et al. [140,141] can control the phase of the transmitted sound wave according to the gradient, so as to realize the abnormal regulation of the transmitted sound wave.

In the past five years, the idea based on MS has achieved many properties of singular regulation of sound waves. The AMS composed of subwavelength Helmholtz resonator arrays can direct the reflected sound waves [142]. The use of MS can make sound waves propagate asymmetrically [143,144,145]. Combining the periodicity of the supercell and the generalized reflection law, when the incident angle exceeds the critical angle, a graded AMS can achieve significant negative reflections [146]. A new type of ultra-thin planar Schroeder diffuser based on the concept of AMS [147] can achieve satisfactory sound diffusion. This has huge application potential in architectural acoustics and related fields. Designing MS using elastic spiral arrays [148,149], by stretching the spiral array along the axial direction can control the band gap, so as to design a new type of acoustic switch. Bok et al. [150] designed an AMS with a thickness of only 1/100 wavelength. The MS is composed of a group of MA, each containing a set of membranes and a cavity filled with air, and it can achieve high-efficiency sound transmission from water to air. Using the AMS phase compensation method, an acoustic invisibility cloak can be realized [151,152,153]. This kind of cloak has simple design, low loss, and has certain application prospects.

The use of sub-wavelength thickness MS to achieve high-efficiency sound absorption has a wide range of application prospects. Ma et al. [154] designed an AMS based on a coupled membrane structure, and used its hybrid resonance state to match the impedance of the structure with the impedance of air to achieve perfect absorption of sound waves. Li et al. [155] designed a metasurface that matches the acoustic impedance of air at the tuning frequency by coupling different resonators and generating mixed resonance modes, which can achieve more than 99% energy absorption at the center frequency of 511 Hz. The use of porous MS and three-dimensional single-ended labyrinth MS can achieve high-efficiency absorption of sound waves in a wide frequency range [156,157]. Jimenez et al. [158] achieved complete quasi-omnidirectional sound absorption by using MS. At present, researchers are mainly concerned with the use of MS to achieve broadband absorption of low-frequency sounds [159,160].

AMS can also achieve super-resolution imaging effects on sound waves [161]. A low-density single-phase hyperlens with a star-shaped lattice structure made of steel has double-negative parameter properties. It can achieve acoustic focusing beyond the diffraction limit [162]. Esfahlani et al. [163] realized the first acoustic dispersion prism based on the unique properties of acoustic transmission line MM, and using the unique physical behavior of acoustic leakage wave radiation. Xie et al. [164] used a two-dimensional metamaterial active phase array as sub-wavelength pixels to achieve acoustic holographic imaging, avoiding complicated circuit design and greatly reducing system complexity. The metamaterial-based hologram can be used as a variety of advanced acoustic waves. A universal platform for operation and signal modulation. Song et al. [165] realized AM with low loss and large refractive index through alternative methods. The fractal method is used to achieve super-resolution imaging, tunneling effect, and excellent flat panel focusing effect in a wide frequency range.

In summary, the development of AM and MS composed of artificial MA or MM has gone from the initial stage “how to design single-negative and double-negative metamaterials” to the current stage of “metamaterials and MS achieve abnormal control of sound waves”. In this development process, the basic unit MA and MM have a very flexible design space, which also provides more possibilities for the regulation of sound waves. In addition, the introduction of some new methods and concepts (such as topological acoustics, etc. [166,167,168,169,170]) has increased the feasibility and practicability of abnormal sound waves. According to the current development trend, it is believed that artificially designed AM and MS can achieve arbitrary adjustment of sound waves according to human needs, and it is expected to transform from basic research to the fields of application.

Although MS show great promise in various application fields, certain challenges lie ahead for their mass deployment and low-cost manufacturing [58,170,171]. Furthermore, MS consist of subwavelength resonators with acoustic properties that are often frequency dispersive. This indicates that the resonant nature of a MS constitutive elements is restricted [172]. Recent research advances indicate that MS are moving toward miniaturization, self-adapting, programming, and digitalization. [173]. In the future, AM and MS are expected to achieve medical high-definition ultrasound imaging, sonar stealth for ships in the water, and effective control of urban noise pollution.

## 4. Conclusions

AM are an artificially structured material with unique properties that cannot be found in natural materials, such as negative refraction, slab focusing, super-resolution imaging, cloaking, inverse Doppler effect, etc. In this article, we introduced a review on the research advances in AM in the recent two decades, and discussed the research achievements in the MA, MM, MA cluster, and MM cluster AM. Finally, we introduced the research status and development trends of AM and MS in the upcoming years.
